# A146 THROAT TROUBLES: CASE REPORT OF LYMPHOCYTIC ESOPHAGITIS IN A CHILD WITH DYSPHAGIA.

**DOI:** 10.1093/jcag/gwae059.146

**Published:** 2025-02-10

**Authors:** I A Alzahrani

**Affiliations:** Gastroenterology, Hepatology and Nutrition, The Hospital for Sick Children, Toronto, ON, Canada

## Abstract

**Background:**

Lymphocytic esophagitis (LE) is a rare and poorly understood inflammatory condition of the esophagus.it is characterized by presence of lymphocyte within esophageal mucosa. It has unique histologic phenotype characterized by increased intraepithelial lymphocytes in the absence or minimal presence of intraepithelial granulocytes. It is unclear whether LE is a unique primary inflammatory esophageal disease or a marker of an underlying esophageal or systemic immune-mediated disease process. Clinical presentations range from asymptomatic to dysphagia, heartburn, chest pain, nausea, vomiting, strictures, and food bolus impaction. Given the rarity of the disease, the incidence is not well known, with one study reporting incidence of LE 0.1% of total esophageal biopsies based on the results of a large pilot study in the United States of more than 40,000 patients.

**Aims:**

To report on a patient with lymphocytic esophagitis who exhibited chronic dysphagia.

**Methods:**

We describe a 6-year-old male, who has a history of coughing and choking with solid foods, as well as dysphagia. Although he has not experienced food impactions, he occasionally vomits undigested food. He was evaluated previously for eosinophilic esophagitis (EoE), with an unremarkable esophagogastroduodenoscopy (EGD). He was diagnosed with gastroesophageal reflux disease (GERD) in his first weeks of life due to projectile vomiting and arching of the back after feeding. He started on proton pump inhibitors (PPIs) at 7 weeks old, continuing until he was 2 years old, with initial vomiting persisting but gradually improving over time.

**Results:**

He had an upper endoscopy again at 6 years which was unremarkable macroscopically. Pathology showed features of lymphocytic esophagitis in upper/mid/distal esophagus and focal basal spongiosis in mid esophagus, no intra-epithelial eosinophils are detected in all esophageal segments. He was started on proton pump inhibitors (PPIs) with good clinical response and improvement of dysphagia and reflux symptoms.

**Conclusions:**

We present a patient with a history of chronic dysphagia and histological findings of LE. LE is a rare subtype of chronic esophagitis, that can include a wide range of clinical presentations. It should be considered in the differential diagnosis of dysphagia or reflux symptoms. Patients with LE can respond to proton PPI therapy and this should be the first line of therapy. However, steroid therapy should be considered for PPI non-responder LE patients. This case raises the importance of heightened awareness regarding the clinical presentation of LE and emphasizes the need for increased recognition of this condition. Although LE seems to have a chronic but benign course, more research is needed to better understand the pathophysiology and natural history of this condition.

**Funding Agencies:**

None

